# Identifying the high-benefit population for weight management-based cardiovascular disease prevention in Japan

**DOI:** 10.1016/j.pmedr.2024.102782

**Published:** 2024-06-04

**Authors:** Sho Tano, Tomomi Kotani, Seiko Matsuo, Takafumi Ushida, Kenji Imai, Hiroaki Kajiyama

**Affiliations:** a Department of Obstetrics and Gynecology, Nagoya University Graduate School of Medicine, Nagoya, Aichi, Japan; b Division of Perinatology, Center for Maternal-Neonatal Care, Nagoya University Hospital, Nagoya, Aichi, Japan

**Keywords:** Annual BMI change, Obesity, Atherosclerosis, Prevention

## Abstract

**Background:**

Cardiovascular-disease (CVD) is the leading cause of death, and the association between obesity and CVD is particularly significant among women. Given the evidence highlighting the significance of weight-gain velosity, we aimed to elucidate its influence on cardio-ankle vascular index (CAVI), a reliable surrogate marker of CVD, and identify the high-benefit population where this influence is most pronounced.

**Methods:**

This multicenter retrospective study used electronic data from annual health checkups for workers in Japan. Individuals who voluntarily measured CAVI in 2019 were included, and weight-gain velosity was defined as the mean BMI gain from 2015 to 2019. Our primary outcome was the relationship between weight-gain velosity and CAVI.

**Results:**

Among 459 individuals, 53 had CAVI ≥ 9. Random forest analysis revealed that age was the most important factor, followed by lipid metabolism, weight-gain velosity, and glucose metabolism, with sex being the least important. Non-linear regression analysis of the effect of age on CAVI ≥ 9 showed the effect was pronounced after age 60, and the trend was greater in women. Among individuals aged 60 or younger, the aOR of weight-gain velosity for CAVI ≥ 9 was significantly positive (aOR 11.95, 95 %CI 1.13–126.27), while it was not significant for those older than 60. The relationship between weight-gain velosity and CAVI provides a new perspective on CVD risk factors. The effects of age, especially after 60, and weight-gain velosity in early- to middle-adulthood on arterial stiffness are emphasized.

**Conclusions:**

These findings underscore the importance of weight management under age 60, especially in women.

## Highlights

• Age, lipid metabolism, and body weight, in that order, were pivotal for arterial stiffness.
• A marked association between age and arterial stiffness was observed after age 60.
• Current BMI and weight gain to date impacted arterial stiffness differently.
• This study addresses the “Obesity Paradox”.
• The best target for weight control to prevent arteriosclerosis was under age 60.

## 1 Introduction

Being overweight and obesity are major risk factors for cardiovascular disease (CVD) (Jia et al., 2019), which is the leading cause of morbidity worldwide (Mortality and Causes of Death, 2015). Although CVD outcomes have improved markedly in recent years, the number of CVD victims due to obesity continues to rise (Shah et al., 2019). It is also known that the association of obesity and CVD is particularly impactful for women: obesity increases the risk of CVD by 1.5-fold in women (Manrique-Acevedo et al., 2020, Wilson et al., 2002). Furthermore, a stronger association between atherosclerosis and CVD mortality has been reported in women compared to men (Coutinho, 2014), and fatal CVD is more likely to occur in women than in men at older ages (di Giosia et al., 2017, Writing Group et al., 2016).

Increased arterial stiffness is recognized to be associated with worse CVD morbidity and mortality (Mitchell et al., 2010). Pulse wave velocity (PWV) is the most commonly used indicator of arterial stiffness, but is essentially influenced by blood pressure (BP) at the time of measurement (Shirai et al., 2011). Recently, the cardio-ankle vascular index (CAVI) has been developed as an arterial stiffness parameter that is not affected by BP (Shirai et al., 2006, Saiki et al., 2020, Otsuka et al., 2014). CAVI has been reported to be associated with CVD development and its severity (Miyoshi et al., 2010, Mcgill, et al., 2002, Nakamura et al., 2009, Saiki et al., 2021, Otsuka et al., 2014). By mitigating the impact of BP, CAVI can evaluate the arterial stiffness attributed to CVD risks beyond hypertension (e.g., obesity) without underestimation (Nagayama et al., 2022a).

Recently, a growing body of evidence has highlighted the clinical significance of the annual body mass index (BMI) change (kg/m^2^/year), alternatively reported as weight change velocity, in addition to the already established risk factors of overweight and obesity (Tano et al., 2021, Esposito et al., 2003, Tano et al., 2022a, Gong et al., 2016, Tano et al., 2022b, Li et al., 2022). Most guidelines for managing body weight to be normal weight (18.5–25.0 kg/m^2^) based on cross-sectional studies, but this approach may be impractical for severely obese patients. Our recent studies suggest that weight gains with age in all ages (Tano et al., 2023), and annual BMI change before conception is related to developing perinatal complications, at risk for CVD in later life, for any BMI individuals (Tano et al., 2022b, Tano et al., 2022a, Tano et al., 2021). We believe that weight management is not an intervention for the condition of overweight/obesity, but for the weight change. However, the association between annual BMI change and CVD risks remains unclear. Based on these findings, we have investigated the association between annual BMI change and CVD risks using CAVI.

Since it is known that suppressing age-related weight gain (0.1–0.2 kg/m^2^/year) is quite challenging (Williams and Wood, 2006), weight management is an intervention that presents a considerable burden for both individuals and healthcare providers, often more than anticipated. Accordingly, it is crucial to concentrate on those who are most likely to benefit from such interventions, which are consistent with the policy of the high-benefit approach (Inoue et al., 2023). Thus, we also aimed to identify the specific group (high-benefit population) where this influence is most pronounced. Additionally, we analyzed gender differences due to the recent focus on sex-specific medicine (Ventura-Clapier et al., 2017).

## 2 Materials and methods

This multicenter retrospective study was conducted at four facilities belonging to the Central Clinic Group in Aichi Prefecture, Japan, using electronic data from annual health checkups for workers at 4,518 companies. Previously, we found that BMI increased linearly from 2015 to 2019, and this trend changed after the COVID-19 pandemic (2020 to 2021) (Tano et al., 2023). Therefore, individuals who had voluntarily measured CAVI in 2019 and for whom it was possible to calculate the annual BMI change from 2015 to 2019 were included in the study.

**Fig. 1:**
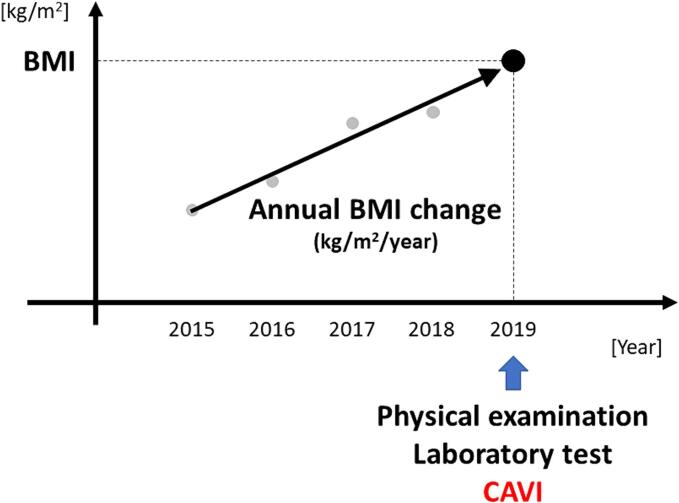
**Overview of the definitions of terms in Japan, 2015**–**2019.** We defined annual annual BMI change as mean BMI change from 2015 to 2019 (red arrow). All parameters used in the analyses including BMI and CAVI were measured in 2019. CAVI, cardio-ankle vascular index; BMI, body mass index. (For interpretation of the references to color in this figure legend, the reader is referred to the web version of this article.)

Using measured height and body weight, BMI was calculated (kg/m^2^). Overweight was defined as BMI of ≥ 25.0 kg/m^2^. Blood was collected from the antecubital vein in the morning after 12 h fasting. All the blood levels were measured according to standard procedures. CAVI was measured using a VaSera VS-1000 (Fukuda Denshi Co Ltd, Tokyo, Japan), according to the method described (Shirai et al., 2006). For the evaluation of CAVI, mean values of the right and left sides were used. We defined annual BMI change as the mean BMI change from 2015 to 2019 (kg/m^2^/year), as shown in Fig. 1. The information on smoking status, anti-diabetic drug use, anti-hyperlipidemic drug use, and antihypertensive drug use is based on the questionnaire.

The primary outcome was the association between annual BMI change and CAVI, and the strength of the association with variables was also examined for CAVI ≥ 9, a known cutoff value for predicting future CVD (Nakamura et al., 2008, Saiki et al., 2020, Sata et al., 2021). Continuous variables are presented as mean ± standard deviation (SD), while categorical variables are expressed as number and percentage (%). The continuous variables were compared using student’s *t*-test or Mann–Whitney *U* test. The categorical variables were compared using the chi-squared test or Fisher’s exact test. With using complete data, we trained a Random Forest (RF) model with CAVI ≥ 9 as the target variable and assessed variable importance using the mean decrease in Gini impurity. Furthermore, the non-linear association between CAVI and annual BMI change was evaluated using generalized additive model (GAM), adjusting for variables selected based on previous studies: age, sex, BMI, hemoglobin (Hb), total cholesterol (T-Chol), high density lipoprotein cholesterol (HDL), triglyceride (TG), fasting plasma glucose (FPG), HbA1c, antidiabetic drug (Anti-DM), and antihyperlipidemic drug (Anti-HL) (Nagayama et al., 2013, Saiki et al., 2021, Shimizu et al., 2014). The study employed both univariable and multivariable logistic regression analyses to compute the crude odds ratios (cORs) and adjusted odds ratios (aORs) of annual BMI change in relation to CAVI ≥ 9. In a sensitivity analysis, multiple linear regression analysis was performed with complementing missing values by multivariate imputation by chained equations using the predictive mean matching function (MICE-PMM, m = 10). All statistical analyses were performed using SPSS software version 28.0 (IBM, USA) and R ver.4.1.3 (https://cran.r-project.org/), and the RF model was implemented using the randomForest ver.4.7–1.1 package in R. Statistical significance was set at two-tailed *p*-value of < 0.05.

This study was approved by the ethics committee of Nagoya University Hospital (2015–0415) in accordance with the guidelines of the Declaration of Helsinki. The requirement for written informed consent was waived due to the retrospective nature of the study.

## 3 Results

### 3.1 Participants

**Fig. 2:**
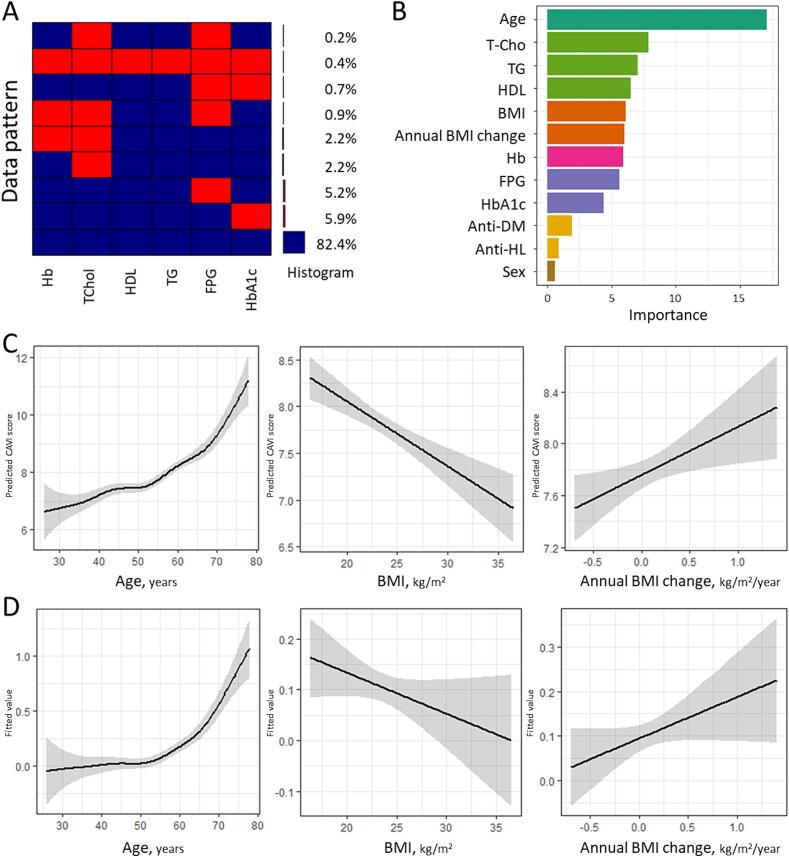
**Non-linear regression analysis on CAVI in Japan, 2015**–**2019.** A. Heatmap shows the distribution of missing data: blue for valid data, red for missing data. The histogram right to the heatmap shows the incidence of each pattern of data. B. Bar plot representing the variable importance obtained from a random forest model for predicting CAVI ≥ 9. Variables are sorted on the y-axis in order of decreasing importance. The color of the bars represents different groups of variables. C. Predicted CAVI (solid-line) with 95 % CI (shaded-area) calculated by a generalized additive model using age, sex, BMI, annual BMI change, smoking status, hemoglobin, total cholesterol, high density lipoprotein cholesterol, triglyceride, fasting plasma glucose, HbA1c, antidiabetic drug, and antihyperlipidemic drug as covariables. D. Fitted value (solid-line) with 95 % CI (shaded-area) of CAVI ≥ 9 calculated by a generalized additive model using age, sex, BMI, annual BMI change, smoking status, hemoglobin, total cholesterol, high density lipoprotein cholesterol, triglyceride, fasting plasma glucose, HbA1c, antidiabetic drug, and antihyperlipidemic drug as covariables. CAVI, cardio-ankle vascular index; BMI, body mass index. (For interpretation of the references to color in this figure legend, the reader is referred to the web version of this article.)

As shown in Table 1, among 459 individuals (men, n = 330; women, n = 129), 53 individuals (11.5 %) had CAVI ≥ 9, and 82.4 % had complete data (Fig. 2A). Regarding 2015 parameters, the group with CAVI ≥ 9 exhibited a lower prevalence of women (11.3 % vs. 30.3 %, *p* = 0.004) and advanced age (58.5 ± 7.2 vs. 48.0 ± 8.6 years, *p* < 0.001), and had higher prevalences of HT (43.4 % vs. 19.7 %, *p* < 0.001) and DM (15.1 % vs. 4.2 %, *p* < 0.001), and lower HDL levels (57.1 ± 13.3 vs. 62.4 ± 17.8 mg/dL, *p* = 0.043). Regarding 2019 parameters, there were significant differences with higher rates of HT (56.6 % vs. 29.1 %, *p* < 0.001), use of anti-HT drugs (35.8 % vs. 18.0 %, *p* = 0.002), DM (15.1 % vs. 4.4 %, *p* = 0.002), and anti-DM drugs (13.2 % vs. 3.2 %, *p* = 0.004). Additionally, elevated systolic and diastolic blood pressure (sBP, 130.9 ± 15.6 vs. 118.8 ± 17.0 mmHg, *p* < 0.001; dBP, 80.3 ± 10.7 vs. 74.7 ± 12.6 mmHg, *p* = 0.002), and reduced HDL levels (56.9 ± 14.7 vs. 62.4 ± 16.5 mg/dL, *p* = 0.022) were observed. No statistically significant differences were noted in BMI (23.7 ± 3.0 vs. 23.6 ± 3.6 kg/m^2^, *p* = 0.814) or annual BMI change (0.10 ± 0.29 vs. 0.09 ± 0.29 kg/m^2^/year, *p* = 0.993).

**Table 1:** Comparison of the characteristics by CAVI evaluated in Japan, 2015 and 2019.

	CAVI ≥ 9 (n = 53)	CAVI < 9 (n = 406)	*p*-value
***Characteristic in 2015***			
**Sex, women**	**6 (11.3)**	**123 (30.3)**	0.004*
**Age, years**	**58.5 ± 7.2**	**48.0 ± 8.6**	<0.001*
BMI, kg/m^2^	23.4 ± 3.0	23.2 ± 3.4	0.807
Overweight	15 (28.3)	111 (27.3)	0.883
Current smoker	11 (20.8)	73 (18.2)	0.647
**HT (%)**	**23 (43.4)**	**80 (19.7)**	<0.001*
Anti-HT durgs (%)	11 (20.8)	46 (11.4)	0.054
**DM (%)**	**8 (15.1)**	**17 (4.2)**	<0.001*
Anti-DM drugs (%)	4 (7.5)	11 (2.7)	0.065
HL (%)	23 (43.4)	149 (36.7)	0.344
Anti-HL drugs (%)	8 (15.1)	31 (7.7)	0.071
**sBP, mmHg**	**130.0 ± 12.7**	**119.9 ± 15.5**	<0.001*
**dBP, mmHg**	**77.6 ± 8.4**	**71.5 ± 12.0**	<0.001*
HR, bpm	58.5 ± 6.4	66.8 ± 7.9	0.161
WBC, ×10^3^/μL	54.3 ± 16.5	54.8 ± 15.3	0.825
Hb, g/dL	14.9 ± 1.1	14.5 ± 1.4	0.126
**Plt, ×10^4^/μL**	**21.9 ± 5.2**	**25.1 ± 4.9**	<0.001*
TChol, mg/dL	203.8 ± 30.5	204.2 ± 34.2	0.932
**HDL, mg/dL**	**57.1 ± 13.3**	**62.4 ± 17.8**	0.043*
TG, mg/dL	120.8 ± 69.2	107.9 ± 76.1	0.251
FPG, mg/dL	99.1 ± 17.3	94.8 ± 17.8	0.104
HbA1c, %	5.7 ± 0.7	5.6 ± 0.7	0.247
Annual BMI change, kg/m^2^/year	0.10 ± 0.29	0.09 ± 0.29	0.993

***Characteristic in 2019***			
**Age, years**	**62.5 ± 7.2**	**52.0 ± 8.6**	<0.001*
BMI, kg/m^2^	23.7 ± 3.0	23.6 ± 3.6	0.814
Overweight	19 (35.8)	123 (30.3)	0.411
Current smoker	9 (17.0)	70 (17.2)	0.962
**HT (%)**	**30 (56.6)**	**118 (29.1)**	<0.001*
**Anti-HT drugs (%)**	**19 (35.8)**	**73 (18.0)**	0.002*
**DM (%)**	**8 (15.1)**	**18 (4.4)**	0.002*
**Anti-DM drugs (%)**	**7 (13.2)**	**13 (3.2)**	0.004*
HL (%)	30 (56.6)	198 (48.8)	0.283
Anti-HL drugs (%)	10 (18.9)	48 (11.8)	0.147
**sBP, mmHg**	**130.9 ± 15.6**	**118.8 ± 17.0**	<0.001*
**dBP, mmHg**	**80.3 ± 10.7**	**74.7 ± 12.6**	0.002*
HR, bpm	75.0 ± 14.5	76.6 ± 11.8	0.712
WBC, ×10^3^/μL	55.3 ± 16.2	54.9 ± 16.9	0.873
Hb, g/dL	14.7 ± 1.0	14.5 ± 1.4	0.543
**Plt, ×10^4^/μL**	**21.9 ± 5.0**	**25.0 ± 5.0**	<0.001*
TChol, mg/dL	202.0 ± 37.3	205.5 ± 32.7	0.491
**HDL, mg/dL**	**56.9 ± 14.7**	**62.4 ± 16.5**	0.022*
TG, mg/dL	122.2 ± 54.9	107.1 ± 61.9	0.093
FPG, mg/dL	99.0 ± 12.2	94.3 ± 17.2	0.068
HbA1c, %	5.7 ± 0.5	5.6 ± 0.6	0.070
CAVI	9.7 ± 0.8	7.5 ± 0.7	−

CAVI, cardio-ankle vascular index; BMI, body mass index; HT, hypertension; DM, diabetes mellitus; HL, hyperlipidemia; sBP, systolic blood pressure; dBP, diastolic blood pressure; HR, heart rate; WBC, white blood cells; Hb, hemoglobin; Plt, platelet; TChol, total cholesterol; HDL, high density lipoprotein cholesterol; TG, triglyceride; FPG, fasting plasma glucose.

*Statistically significant.

### 3.2 Association between annual BMI change and CAVI

In the RF model, as depicted in Fig. 2B, age held the highest importance with a value of 17.09, followed by variables related to lipid metabolism (T-Cho 7.86 and HDL 7.04), body weight (BMI 6.09 and annual BMI change 5.98), hemoglobin levels (Hb 5.87), and glucose metabolism (FPG 5.60 and HbA1c 4.37). Sex held the lowest importance, with a value of 0.57.

**Fig. S1:**
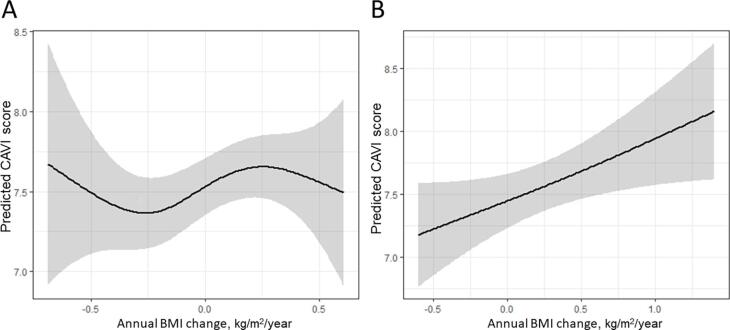
**Non-linear regression analysis on CAVI classified with overweight status in Japan, 2015–2019.** Predicted CAVI (solid-line) with 95% CI (shaded-area) calculated by a generalized additive model using age, sex, BMI, annual BMI change, smoking status, hemoglobin, total cholesterol, high density lipoprotein cholesterol, triglyceride, fasting plasma glucose, HbA1c, antidiabetic drug, and antihyperlipidemic drug as covariables. A for non-overweight participants and B for overweight participants.

The results of the generalized additive models predicting CAVI score using complete data sets are displayed in Fig. 2C. The analysis revealed the relationship between age and CAVI was not completely linear but positive. There was a significant negative linear association between BMI and CAVI, as well as a significant positive linear association between annual BMI change and CAVI. The linear regression analyses on CAVI (Model1 of Table 2) indicated positive coefficients for age (B = 0.06, Beta = 0.55, *p* < 0.001), annual BMI change (B = 0.35, Beta = 0.10, *p* = 0.021), and TG (B = 0.03, Beta = 0.18, *p* < 0.001), while the coefficient for BMI was negative (B = -0.07, Beta = -0.24, *p* < 0.001). Sex (women) had negative coefficients (B = -0.26, Beta = -0.11, *p* = 0.039). Further sensitivity analysis with the addition of blood pressure data did not alter the findings (Model 2 of Table 2). The sensitivity analysis that involved MICE-PMM to complement the missing data did not alter the primary findings (Table S1). Subgroup analysis according to whether or not the patient was overweight in 2019 showed that for annual BMI change that settled in the non-overweight range (Fig. S1A), there was no clear positive relationship between annual BMI change and CAVI. In contrast, for individuals who were overweight in 2019, annual BMI change was positively associated with CAVI (Fig. S1B). Table S2 shows the results of the linear regression analyses using 2015 parameters and annual BMI change as covariates, and although not statistically significant for the annual BMI change results, B = 0.23 for Model 1 and B = 0.19 for Model 2.

**Table 2:** Multiple linear regression analysis on CAVI in Japan, 2019.

Variables in 2019	Model 1*					Model 2^§^			
	Unstandardized Coefficients		Standardized Coefficient	*p*-value		Unstandardized Coefficients		Standardized Coefficient	*p*-value
	B	SE	Beta			B	SE	Beta	
**Age, years**	**0.06**	**0.01**	**0.55**	<0.001*		**0.06**	**0.01**	**0.50**	<0.001*
**Sex, women**	**−0.26**	**0.13**	**−0.11**	0.039*		**−0.30**	**0.12**	**−0.13**	0.014*
**BMI, kg/m^2^**	**−0.07**	**0.01**	**−0.24**	<0.001*		**−0.10**	**0.02**	**−0.34**	<0.001*
**Annual BMI change, kg/m^2^/year**	**0.35**	**0.15**	**0.10**	0.021*		**0.33**	**0.15**	**0.09**	0.026*
TChol^†^, mg/dL	−0.01	0.01	−0.04	0.369		−0.02	0.01	−0.05	0.277
HDL^†^, mg/dL	−0.01	0.04	−0.02	0.717		−0.02	0.03	−0.04	0.521
**TG**^†^**, mg/dL**	**0.03**	**0.01**	**0.18**	<0.001*		**0.03**	**0.01**	**0.17**	0.001*

CAVI, cardio-ankle vascular index; SE, standard error; BMI, body mass index, TChol, total cholesterol; HDL, high density lipoprotein cholesterol; TG, triglyceride.

*Model 1: Adjusted for age, sex, BMI, annual BMI change, hemoglobin, total cholesterol, high density lipoprotein cholesterol, triglyceride, fasting plasma glucose, HbA1c, antidiabetic drug, and antihyperlipidemic drug.

^§^Model 2: Model 1 + systolic blood pressure + diastolic blood pressure + Anti-hypertensive drug.

^†^per 10 mg/dL increment. *Statistically significant.

**Fig. S2:**
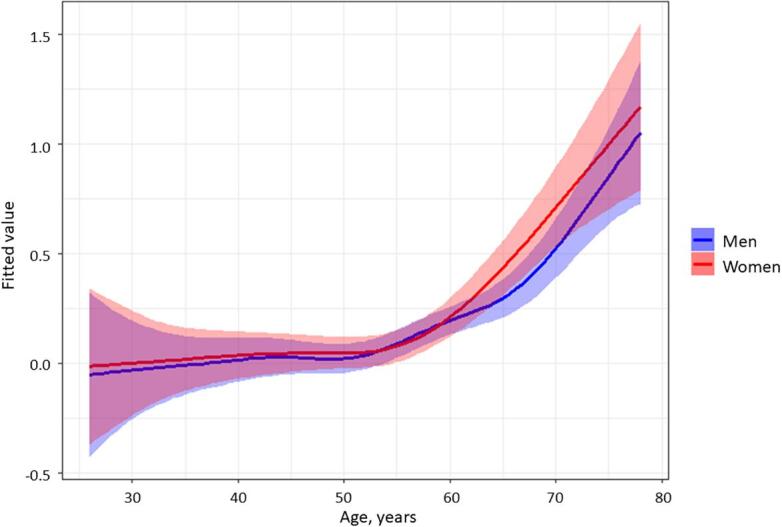
**Sex-stratified non-linear regression analysis on CAVI in Japan, 2015–2019.** Sex-stratified analyses predicting fitted value (solid-line) with 95% CI (shaded-area) of CAVI ≥9 calculated by a generalized additive model using age, sex, BMI, annual BMI change, smoking status, hemoglobin, total cholesterol, high density lipoprotein cholesterol, triglyceride, fasting plasma glucose, HbA1c, antidiabetic drug, and antihyperlipidemic drug as covariables.

Further non-linear regression analysis was performed to assess the impact of age on CAVI ≥ 9, revealing that the effects of aging were particularly pronounced after the age of 60 (Fig. 2D). Subsequently, sex-stratified analyses indicated that the positive association between age and CAVI ≥ 9 was similar for both sexes until the age of 60 (Fig. S2). But after age 60, the effect of age trended stronger for women than for men, although it was not statistically significant. The association between BMI and CAVI ≥ 9 was negative and linear, as well as a significant positive linear association between annual BMI change and CAVI, although the 95 % confidence interval (CI) was slightly wide (Fig. 2D). Table 3 displays the findings of the multivariable logistic regression analyses with CAVI ≥ 9 as the dependent variable, stratified according to age groups: young to middle adulthood (≤60 years old) and senior group (>60 years old). In the former group, a significant positive association was observed between annual BMI change and CAVI ≥ 9 (aOR 11.19, 95 %CI 1.03–121.69). Conversely, in the senior group, age was the sole variable significantly associated with CAVI ≥ 9 (aOR 1.44, 95 %CI 1.17–1.76), while the association between annual BMI change and CAVI ≥ 9 was not statistically significant (aOR 0.97, 95 %CI 0.06–15.87). With regard to sex, the association with CAVI ≥ 9 was initially statistically significant (cOR 0.21, 95 %CI 0.48–0.92), but failed to maintain this level of significance after the adjustment (cOR 0.27, 95 %CI 0.04–1.76). Regarding the covariables entered for adjustment, their distribution by sex revealed clear differences in distribution for lipid metabolism (Table S3): women have higher HDL (74.2 ± 15.0 mg/dL vs. 56.8 ± 14.1, *p* < 0.001) and lower TG (79.2 ± 43.9 mg/dL vs. 120.6 ± 63.2, *p* < 0.001). Regarding T-Chol, there was a slightly higher trend among women, although it was not statistically significant (210.0 ± 35.7 vs. 203.2 ± 32.1 mg/dL, *p* = 0.057).

**Table 3:** Univariable and multivariable logistic regression analyses on CAVI ≥ 9 in Japan, 2019.

Variables in 2019	Young to middle adulthood (≤60 years old) (n = 357)				Senior (>60 years old) (n = 102)			
	cOR	(95 %CI)	aOR^§^	(95 %CI)	cOR	(95 %CI)	aOR^§^	(95 %CI)
Age, years	1.27*	**(1.13**–**1.42)**	1.32*	**(1.15**–**1.52)**	1.12*	**(1.02**–**1.23)**	1.44*	**(1.17**–**1.76)**
Sex, women	0.21*	**(0.48**–**0.92)**	0.27	(0.04–1.76)	0.77	(0.22–2.62)	1.48	(0.95–22.95)
BMI, kg/m^2^	0.98	(0.86–1.11)	0.85	(0.68–1.08)	0.98	(0.86–1.10)	0.97	(0.75–1.27)
Annual BMI change, kg/m^2^/year	1.63	(0.39–6.74)	11.95*	**(1.13**–**126.27)**	0.53	(0.10–2.87)	0.97	(0.06–15.87)
TChol^†^, mg/dL	1.03	(0.91–1.18)	1.00	(0.83–1.20)	0.99	(0.85–1.14)	0.98	(0.75–1.28)
HDL^†^, mg/dL	0.93	(0.71–1.21)	0.96	(0.62–1.49)	0.79	(0.56–1.12)	0.87	(0.38–2.00)
**TG**^†^**, mg/dL**	1.01	(0.98–1.05)	1.00	(0.96–1.05)	1.03	(0.95–1.12)	0.93	(0.77–1.13)

CAVI, cardio-ankle vascular index; cOR, crude odds ratio; aOR, adjusted odds ratio; 95 %CI, 95 % confidence interval; BMI, body mass index, TChol, total cholesterol; HDL, high density lipoprotein cholesterol; TG, triglyceride.

^§^aORs were adjusted for age, sex, BMI, annual BMI change, hemoglobin, total cholesterol, high density lipoprotein cholesterol, triglyceride, fasting plasma glucose, HbA1c, antidiabetic drug, and antihyperlipidemic drug.

^†^per 10 mg/dL increment. *Statistically significant.

## 4 Discussion

**Fig. S3:**
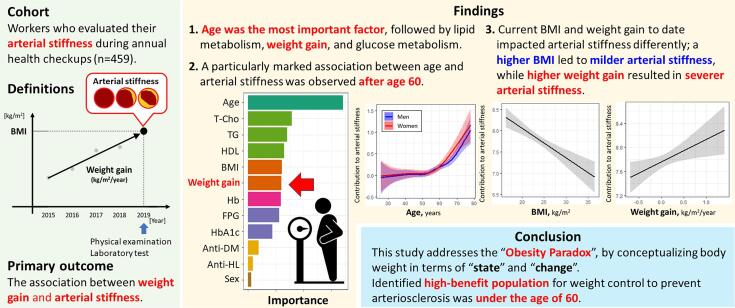
**Graphic abstract**.

This study provides novel insights into the determinants of arterial stiffness as measured by CAVI. First, age was the most important factor regarding CAVI, followed by lipid metabolism and weight-related indicators. The relationship between age and CAVI was particularly pronounced after age 60, and it tended to be stronger in women than in men after age 60. Second, a significant positive linear association was shown between annual BMI change and CAVI, while BMI showed a negative association with CAVI. Third, annual BMI change was associated with CAVI ≥ 9 especially in young to middle adulthood but not in senior individuals. Finally, sex was the least important with no independent association with CAVI. This result suggests that both sexes with the same profile are at equal risk (summarized in Fig. S3).

Previously, a converse relationship between BMI and CAVI is well-established (Nagayama et al., 2017, Nagayama et al., 2022b, Nagayama et al., 2020, Kabutoya et al., 2020). This inverse association is in line with the obesity paradox, which suggests that overweight and obese CVD patients experience favorable outcomes (Gruberg et al., 2002), but there has been a discrepancy regarding the association between obesity and CVD development. Our study also observed a negative association between BMI and CAVI, consistent with this topic. However, the present study provides novel evidence that annual BMI change may have a harmful impact on arterial stiffness, which is in line with the well-established association between obesity and CVD development. Furthermore, it was shown that there are more patients with HT in the CAVI ≥ 9 group, and consequently, the mean sBP and dBP are also higher in this group. However, even in the multivariable analysis (as shown in Table 2 Model 2), which included BP as a covariate, annual BMI change was an independent associated factor of CAVI ≥ 9. Thus, the influence of higher sBP and sBP on the results is likely to be marginal. Moreover, our findings indicate a significant association between annual BMI change and CAVI, particularly during early to middle adulthood. This finding is consistent with previous research demonstrating that weight gain during this period is associated with increased risk of future CVD (Jia et al., 2019). Our subgroup analysis, examining whether weight gain within the non-overweight range significantly increases arterial stiffness, resulted in wide 95 % CIs, preventing us from reaching a definitive conclusion; further investigation is required.

The current study also indicated a robust association between age and CAVI, and further demonstrated a curvilinear trend with a flatter curve at younger ages and a steep increase in arterial stiffness after approximately 60 years of age. A similar curvilinear trend has been reported for the Agatston coronary artery calcium score, which indicates the degree of calcification of coronary arteries, a process of atherosclerosis (McClelland et al., 2006, Alexopoulos and Raggi, 2009). These results underscore the importance of implementing different preventive measures for atherosclerosis in younger and older populations, and suggest that weight management may be a meaningful strategy for younger individuals.

In the analysis conducted using RF, not only TG but also TChol and HDL showed high importance for arterial stiffness. However, in the multiple linear regression analysis, only TG was identified as a significant variable. This difference may be due to the linear regression focusing on the independent associations of each variable, whereas the intricate interactions between factors such as age, weight changes, and sex with lipid metabolism (Pavlovska et al., 2020, Nagayama et al., 2018, Joyce et al., 2014) can be more reflected in the RF analysis.

Identification of high-benefit populations can be expected to most efficiently implement a population approach with weight control, and also provide an opportunity for those who do not fall into the high-benefit population to focus their efforts on preventive measures other than weight control. To improve the prognosis of CVD in women in older ages, weight control under age 60 can be effective as a population approach. Weight management during this period for women is also reasonable for risk management of hypertensive disorders of pregnancy (HDP) (Tano et al., 2021), a well-known sex-specific risk for CVD (Yang et al., 2023). In this study, the frequency of a CAVI ≥ 9 was low among women. Our sex-specific analysis suggested that this could be due to women exhibiting profiles that may be protective against arteriosclerosis, such as lower BMI and lower TG. Additionally, our analysis using RF indicated that sex had the lowest importance, suggesting that being female may not necessarily mitigate the risk in individuals with parameters conducive to atherosclerosis.

Our study has certain limitations that should be acknowledged. Firstly, this study did not assess the effect of intervention-induced weight loss on CAVI, but instead compared individuals who lost weight spontaneously and those who did not. Further investigation is necessary to evaluate the impact of intervention-induced weight loss on arterial stiffness. Secondly, the generalizability of this study might be limited due to the inclusion of only workers whose mean BMI change from 2015 to 2019 was available. Individuals who were unable to undergo annual health checkups due to various reasons, such as unemployment or illness during the period, were excluded from the analysis. However, following individuals to assess measured weight changes helped to improve internal validity. Thirdly, it should be noted that the data collected other than the measured parameters relied on self-completed questionnaires. Due to the limited scope of the questionnaire, detailed information on treatment was not available. However, it is important to note that the data collected through the annual health checkups was conducted under the Japanese Law, Industrial Safety and Health Act, and therefore considered reliable. Finally, the relatively small number of women and the lack of data on obstetrics and menopause precluded more detailed sex-stratified analyses. If we could have conducted a detailed examination for each sex in this analysis, we might have been able to provide further valuable evidence for gender-specific medicine. However, the result that sex was not an independent associated factor, suggests that women are also subjects in need of proactive weight management.

## 5 Conclusions

In conclusion, age was identified as the predominant factor influencing CAVI, with a particularly marked association observed after age 60, especially in women. Our findings reveal that annual BMI change, particularly under age 60, negatively affects arterial stiffness. These findings emphasize the importance of the targeted approach to CVD prevention.

## Funding

The work was supported by the Japan Society for the Promotion of Science Grant-in-Aid for Scientific Research (Grant Number: 23K19800 for Sho Tano).

## CRediT authorship contribution statement

**Sho Tano:** Writing – original draft, Investigation, Funding acquisition, Formal analysis, Conceptualization. **Tomomi Kotani:** Writing – original draft, Methodology, Investigation, Data curation, Conceptualization. **Seiko Matsuo:** Writing – review & editing, Validation, Investigation. **Takafumi Ushida:** Writing – review & editing, Validation, Investigation. **Kenji Imai:** Writing – review & editing, Validation, Investigation. **Hiroaki Kajiyama:** Writing – review & editing, Supervision.

## Declaration of competing interest

The authors declare that they have no known competing financial interests or personal relationships that could have appeared to influence the work reported in this paper.

## Data Availability

Some or all datasets generated during and/or analyzed during the current study are not publicly available but are available from the corresponding author on reasonable request, subject to approval from the Central Clinic Group.
