# Supplementary material for: Identifying the high-benefit population for weight management-based cardiovascular disease prevention in Japan
Source: Prev Med Rep. 2024 Jun 4;43:102782. doi: 10.1016/j.pmedr.2024.102782 (PMC11257143; doi:10.1016/j.pmedr.2024.102782)
Supplement: Supplementary Data 1 [file mmc1.docx]

Table S1. Multiple linear regression analysis on CAVI with predictive miss-matching data imputation in Japan, 2015 and 2019.

| Variables in 2019 | Unstandardized  Coefficients | | *p*-value |
| --- | --- | --- | --- |
|  | B | SE |  |
| **Age, years** | **0.07** | **0.00** | **<0.001*** |
| **Sex, women** | **-0.24** | **0.12** | **0.041*** |
| **BMI, kg/m^2^** | **-0.06** | **0.01** | **<0.001*** |
| **Annual BMI change, kg/m^2^/year** | **0.28** | **0.13** | **0.041*** |

CAVI, cardio-ankle vascular index; SE, standard error; BMI, body mass index,

Adjusted for age, sex, smoking status, BMI, annual BMI change, hemoglobin, total cholesterol, high density lipoprotein cholesterol, triglyceride, fasting plasma glucose, HbA1c, antidiabetic drug, and antihyperlipidemic drug.

*Statistically significant

Table S2. Multiple linear regression analysis on CAVI in 2019 using variables in 2015 as covariables in Japan.

| Variables in 2015 | Model 1* | | | |  | Model 2^§^ | | | |
| --- | --- | --- | --- | --- | --- | --- | --- | --- | --- |
|  | Unstandardized  Coefficients | | Standardized  Coefficient | *p*-value |  | Unstandardized  Coefficients | | Standardized  Coefficient | *p*-value |
|  | B | SE | Beta |  |  | B | SE | Beta |  |
| Age, years | **0.06** | **0.01** | **0.57** | **<0.001*** |  | **0.05** | **0.01** | **0.50** | **<0.001*** |
| Sex, women | -0.10 | 0.14 | -0.05 | 0.462 |  | -0.10 | 0.16 | -0.05 | 0.525 |
| BMI, kg/m^2^ | **-0.08** | **0.02** | **-0.29** | **<0.001*** |  | **-0.11** | **0.02** | **-0.36** | **<0.001*** |
| Annual BMI change, kg/m^2^/year | 0.23 | 0.15 | 0.07 | 0.120 |  | 0.19 | 0.16 | 0.06 | 0.238 |
| TChol^†^, mg/dL | -0.01 | 0.02 | -0.03 | 0.639 |  | -0.00 | 0.02 | -0.00 | 0.965 |
| HDL^†^, mg/dL | -0.07 | 0.04 | -0.13 | 0.053 |  | -0.06 | 0.05 | -0.10 | 0.205 |
| TG^†^, mg/dL | 0.01 | 0.01 | 0.10 | 0.112 |  | **0.02** | **0.01** | **0.14** | **0.045*** |

CAVI, cardio-ankle vascular index; SE, standard error; BMI, body mass index, TChol, total cholesterol; HDL, high density lipoprotein cholesterol; TG, triglyceride.

*Model 1: Adjusted for age, sex, BMI, annual BMI change, hemoglobin, total cholesterol, high density lipoprotein cholesterol, triglyceride, fasting plasma glucose, HbA1c, antidiabetic drug, and antihyperlipidemic drug.

^§^Model 2: Model 1 + systolic blood pressure + diastolic blood pressure + Anti-hypertensive drug.

^†^per 10 mg/dL increment. *Statistically significant.

Table S3. Comparison of the characteristics by sex in Japan, 2015 and 2019.

| Variables in 2019 | Women  n=129 | Men  n=330 | *p*-value |
| --- | --- | --- | --- |
| Age, years | 52.4 ± 8.4 | 53.5 ± 9.3 | 0.229 |
| **BMI, kg/m^2^** | **22.0 ± 3.8** | **24.3 ± 3.2** | **<0.001*** |
| Annual BMI change, kg/m^2^/year | 0.13 ± 0.29 | 0.08 ± 0.29 | 0.100 |
| **Current smoker** | **6 (4.7)** | **73 (22.1)** | **<0.001*** |
| Anti-hypertensive drug (%) | 19 (14.7) | 73 (22.1) | 0.075 |
| **Anti-diabetic drug (%)** | **1 (0.8)** | **19 (5.8)** | **0.019*** |
| Anti-hyperlipidemic drug (%) | 17 (13.2) | 41 (12.4) | 0.827 |
| **sBP, mmHg** | **117.2 ± 20.5** | **121.3 ± 15.7** | **0.023*** |
| **dBP, mmHg** | **70.2 ± 13.4** | **77.3 ± 11.5** | **<0.001*** |
| WBC, ×10^3^/μL | 54.8 ± 19.2 | 55.1 ± 15.9 | 0.861 |
| **Hb, g/dL** | **13.2 ± 1.2** | **15.1 ± 1.0** | **<0.001*** |
| **Plt, ×10^4^/μL** | **26.1 ± 6.0** | **24.2 ± 4.7** | **<0.001*** |
| T-Chol, mg/dL | 210.0 ± 35.7 | 203.2 ± 32.1 | 0.057 |
| **HDL, mg/dL** | **74.2 ± 15.0** | **56.8 ± 14.1** | **<0.001*** |
| **TG, mg/dL** | **79.2 ± 43.9** | **120.6 ± 63.2** | **<0.001*** |
| **FPG, mg/dL** | **89.9 ± 9.3** | **96.7 ± 18.5** | **<0.001*** |
| HbA1c, % | 5.5 ± 0.4 | 5.6 ± 0.6 | 0.083 |
| **CAVI** | **7.4 ± 0.8** | **7.9 ± 1.1** | **<0.001*** |

CAVI, cardio-ankle vascular index; BMI, body mass index; sBP, systolic blood pressure; dBP, diastolic blood pressure; Hb, hemoglobin; Plt, platelet; TChol, total cholesterol; HDL, high density lipoprotein cholesterol; TG, triglyceride; FPG, fasting plasma glucose.*Statistically significant.
